# Neural Organization of A3 Mushroom Body Extrinsic Neurons in the Honeybee Brain

**DOI:** 10.3389/fnana.2018.00057

**Published:** 2018-08-03

**Authors:** Hanna Zwaka, Ruth Bartels, Bernd Grünewald, Randolf Menzel

**Affiliations:** ^1^Institute of Neurobiology, Free University Berlin, Berlin, Germany; ^2^Molecular and Cellular Biology, Harvard University, Cambridge, MA, United States; ^3^Institut für Bienenkunde Oberursel, Goethe University Frankfurt, Frankfurt, Germany; ^4^Bernstein Center for Computational Neuroscience, Berlin, Germany

**Keywords:** GABA, honeybee, mushroom bodies, memory, feedback neurons, mushroom body extrinsic neurons

## Abstract

In the insect brain, the mushroom body is a higher order brain area that is key to memory formation and sensory processing. Mushroom body (MB) extrinsic neurons leaving the output region of the MB, the lobes and the peduncle, are thought to be especially important in these processes. In the honeybee brain, a distinct class of MB extrinsic neurons, A3 neurons, are implicated in playing a role in learning. Their MB arborisations are either restricted to the lobes and the peduncle, here called A3 lobe connecting neurons, or they provide feedback information from the lobes to the input region of the MB, the calyces, here called A3 feedback neurons. In this study, we analyzed the morphology of individual A3 lobe connecting and feedback neurons using confocal imaging. A3 feedback neurons were previously assumed to innervate each lip compartment homogenously. We demonstrate here that A3 feedback neurons do not innervate whole subcompartments, but rather innervate zones of varying sizes in the MB lip, collar, and basal ring. We describe for the first time the anatomical details of A3 lobe connecting neurons and show that their connection pattern in the lobes resemble those of A3 feedback cells. Previous studies showed that A3 feedback neurons mostly connect zones of the vertical lobe that receive input from Kenyon cells of distinct calycal subcompartments with the corresponding subcompartments of the calyces. We can show that this also applies to the neck of the peduncle and the medial lobe, where both types of A3 neurons arborize only in corresponding zones in the calycal subcompartments. Some A3 lobe connecting neurons however connect multiple vertical lobe areas. Contrarily, in the medial lobe, the A3 neurons only innervate one division. We found evidence for both input and output areas in the vertical lobe. Thus, A3 neurons are more diverse than previously thought. The understanding of their detailed anatomy might enable us to derive circuit models for learning and memory and test physiological data.

## Introduction

**Figure 1:**
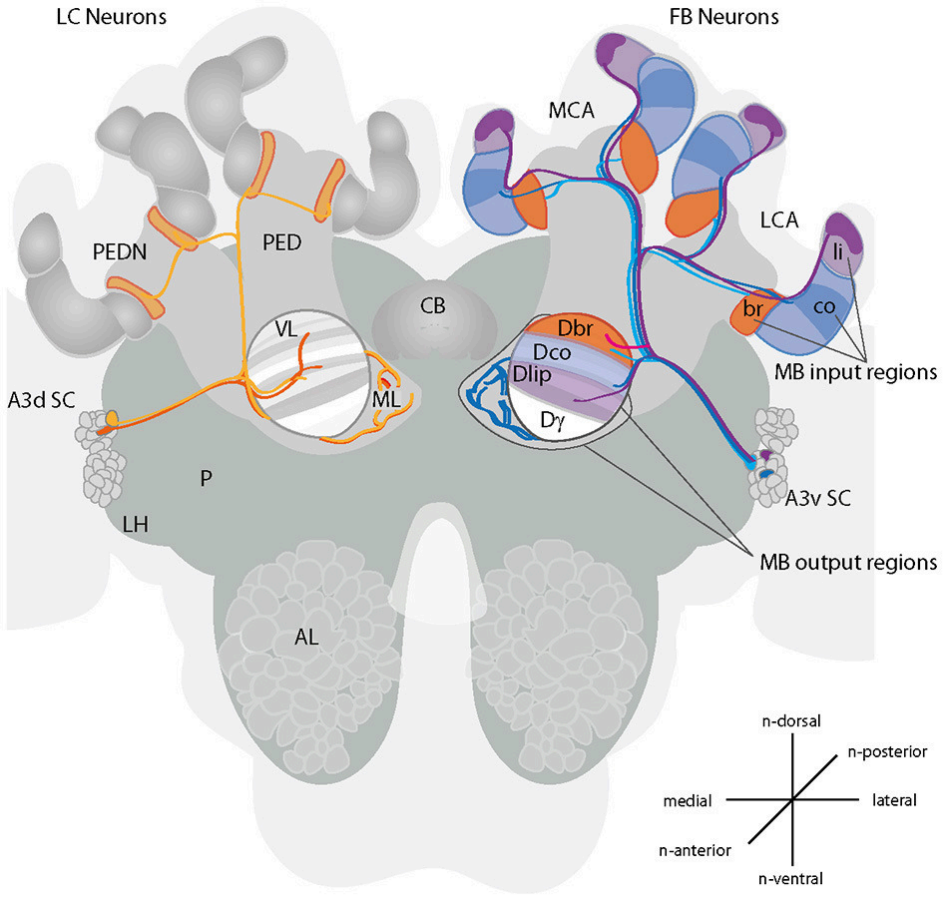
Projections of A3 neurons in the honeybee brain. A schematic drawing of the main innervation types of A3 neurons in the mushroom body (MB) and vertical lobe. Left side of the brain: A3 lobe connecting (LC) neurons connect divisions of the vertical lobe with the medial lobe (orange) or the lobes with the neck region of the peduncle (yellow). In the vertical lobe, neurons connect to multiple divisions. Right side of the brain: A3 feedback neurons (FB) connect calyx-corresponding divisions in the vertical lobe consisting of the lip, collar or basal ring (blue to pink). Depicted are in lighter colors the innervated subcompartments lip, basal ring and collar. The darker areas show the partial innervation by A3 neurons described here. Feedback neurons can connect to the same or different divisions in the vertical lobe as in the MB calyx. There are at least four different A3_FB_ neuron types (depicted in blue, light blue, pink and lilac). Three types connect one calyx region with the corresponding region in the vertical lobe. One type connects the lip region with the division of the basal ring in the vertical lobe (pink). For a schematic overview of innervation in the medial lobe see Figure 3. Cells according to Grünewald (1999b): F1/F3: light blue, F2: pink, F4: blue. A3d SC, dorsal A3 soma cluster; A3v SC, ventral A3 soma cluster; AL, antennal lobe; br, basal ring; CB, central body; co, collar; Dbr, division corresponding to the basal ring; Dco, division corresponding to the collar; Dli, division corresponding to the lip; Dγ, Gamma division; LCA, lateral calyx; FB, feedback; LH, lateral horn; LC, lobe connecting; li, lip; MB, mushroom body; MCA, medial calyx; ML, medial lobe; P, protocerebrum; PED, peduncle; PEDN, peduncle neck; VL, vertical lobe. Prefix “n-” indicates directions that are based on the neuraxis (Ito et al., 2014).

The mushroom body (MB) in the insect brain is key to memory formation and sensory processing (Heisenberg, 2003; Menzel, 2014; Aso and Rubin, 2016). This paired higher-order brain neuropil is thought to integrate multimodal sensory input and direct it to other protocerebral neuropils (Rybak and Menzel, 1993). In the honeybee, each MB consists of four main sub-domains: the two calyces, the medial lobe, and the vertical lobe (Figure 1). The calyces and the lobes are connected by the peduncle consisting of axons of the MB intrinsic neurons, the Kenyon cells (KC). Each calyx is further subdivided into three concentric neuropils: the lip, the collar, and the basal ring, each receiving sensory input (Mobbs, 1982, 1984; Abel et al., 2001; Gronenberg, 2001; Gronenberg and Lopez-Riquelme, 2004; Zwaka et al., 2016). In the calyces, sensory projection neurons feed information primarily on to KCs (Ganeshina and Menzel, 2001). The main output regions of KCs are the vertical lobe (VL) and medial lobe (ML). KC are arranged in such a way that each calycal subcompartment is presented in a separated layer in the vertical lobe, the peduncle, and the medial lobe (Mobbs, 1982; Rybak and Menzel, 1993; Strausfeld, 2002). In these lobes KCs connect to a variety of MB extrinsic neurons including a group of about 110 GABAergic neurons also called A3 neurons (Bicker et al., 1988; Rybak and Menzel, 1993). The inhibitory neurotransmitter GABA is known to be important for specific forms of learning in honeybees (Raccuglia and Mueller, 2013, 2014; Boitard et al., 2015). In bees, several studies have shown that MB extrinsic neurons change their response properties in the course of learning, indicating involvement in processing of sensory information, learning, memory formation and memory retrieval (Mauelshagen, 1993; Reviewed by Hammer, 1997; Grünewald, 1999a; Haehnel and Menzel, 2010, 2012; Filla and Menzel, 2015). Recently, a study shed light on the GABAergic connectivity of the MB alpha lobe in flies (Takemura et al., 2017) that include feedback neurons (Tanaka et al., 2008; Liu and Davis, 2009; Lin et al., 2014; Takemura et al., 2017). Inhibitory feedback neurons are also found in locusts (Leitch and Laurent, 1996; Papadopoulou et al., 2011) and cockroaches (Weiss, 1974; Nishino and Mizunami, 1998; Yamazaki et al., 1998; Strausfeld and Li, 1999; Takahashi et al., 2017).

The 110 A3 neurons in bees, can be divided in two main groups: (1) about half of them are restricted to the medial lobes, the vertical lobes and the peduncle (here referred to as lobe connecting neurons - A3_LC_); whereas the other half (2) project from the medial and vertical lobes via the peduncle to the calyx (here referred to as feedback neurons - A3_FB_) (Gronenberg, 1987; Rybak and Menzel, 1993; Grünewald, 1999b).

A morphological analysis of A3_FB_ in brain slices using cobalt staining and light microscopy suggests that each individual cell homogenously innervates only one calycal subcompartment (the lip, the collar or the basal ring) (Grünewald, 1999b). A3_FB_ neurons also innervate areas in the vertical and medial lobe. As mentioned above, these areas in the lobes have corresponding areas in the calyces. Corresponding here refers to zones that are innervated by the same KCs in the calyces and the lobes. In the following we will call the lip, basal ring, and collar corresponding areas, lip, basal ring, and collar divisions of the respective lobe. Four different types of feedback A3 neurons have been distinguished depending on their innervation areas within the calyces and in the vertical lobe (F1-F4, Figure 1): The basal ring and the collar are innervated by A3_FB_ neurons that also innervate the calyx-corresponding vertical lobe zones (FN1 and FN3- basal ring, FN4- collar). The lip, however is only innervated from a noncorresponding vertical lobe zone that corresponds to the basal ring zone in the calyx (FN2) (Grünewald, 1999b). Each A3 neuron connects to a large proportion of KCs (Grünewald, 1999b).

Anatomical studies on A3 cells have been rare (Takahashi et al., 2017). A detailed anatomy of GABAergic innervation in the MB in honeybees could help to derive a circuit model in order to test and understand physiological data. For example, combining anatomical knowledge about the inhibitory feedback circuit in *Drosophila* larvae with imaging data helped to understand olfactory selectivity in KCs (Masuda-Nakagawa et al., 2014).

In this study, we used confocal imaging of single and multiple A3 neurons which allows for a whole brain analysis of single cell anatomy. With this technique, a more detailed analysis of A3 anatomy was possible and allowed for refining the knowledge about A3_FB_ neurons and defining new subtypes of A3 neurons. Our data shows that A3 feedback neurons innervate the calyx subcompartments with different, more restricted innervation patterns, than previously assumed. For the first time we are describing anatomical details of A3 lobe connecting neurons and compare their innervation in the different MB areas to those of A3 feedback cells. We can show that both types of A3 cells connect corresponding zones in the neck of the peduncle, the medial lobe and the vertical lobe and found evidence for both input and output areas in the vertical lobe.

## Methods

Worker honeybees (*Apis mellifera carnica*) were immobilized by cooling and afterwards were harnessed in plastic tubes. A window was cut into the head capsule between the compound eyes. To prevent movements of the brain, the proboscis and the mandibles were expanded, and the abdomen was gently squeezed. Antennae were fixated with paraffin wax. Around the injection side, head glands, tracheae and the neural sheet covering the frontal surface of the brain were gently removed.

For intracellular dye filling of A3 neurons, electrodes (borosilicate glass capillaries with filament with an o.d. 1.0 mm, i.d. 0.53 and 75 mm length, Hilgenberg GmbH, Malsfeld, Germany) were pulled with a laser-based micropipette puller P-2000 (Sutter Instruments Corp., Novato, CA). Electrode tips were filled with either 5–10% NeurobiotinTM (Vector Laboratories Inc., Ontario, CA) or 4% tetramethylrodamine-biotin dextran (TMR, 3000 MW, Microruby, MoBiTec, Göttingen, Germany) diluted in 0.2 M potassium acetate. Electrodes were inserted into the vertical lobe. Spiking neurons were detected at a depth ranging from 20 to 150 μm and the dye was injected iontophoretically using depolarizing pulses (1–2 Hz, 0.2 s duration, 2–4 nA, 5–20 min; Intra 767, World Precision Instruments, Berlin, Germany). The dye diffused for at least 4 h.

### Histochemistry

For intracellular staining, brains were dissected in bee physiological saline solution (NaCl (130 mM), KCl (6 mM), MgCl_2_ (4 mM), CaCl_2_ (5 mM), glucose (25 mM), sucrose (170 mM) adjusted to pH 6.7 using diluted HCL) and fixed overnight at 4°C in 4% paraformaldehyde (PFA, Roth, Karlsruhe, Germany) in 0.1 M phosphate-buffered saline saline (PBS; NaCl (37 mM), KCl (2.7 mM), Na_2_HPo_4_ (8 mM), KH_2_Po_4_ (1.4 mM), adjusted to pH 6.7 using diluted HCL). Brains were washed three times in PBS for 10 min. If neurons were filled with Neurobiotin, brains were preincubated at room temperature for 2 h in 0.3% Triton X (Sigma-Aldrich, München, Germany) in PBS and incubated over night at 4°C in Streptavidin-Cy5 (Dianova, Hamburg, Germany) and sodium azide (0.005%). Lucifer Yellow (Lucifer Yellow CH dilithium salt, Sigma-Aldrich, München, Germany, 1:100 in PBS, respectively) was added for neuropil staining. On the next day, the brains were washed in PBS (for 15, 30, and 45 min) before they were dehydrated in an ascending ethanol series (each 10 min in 50, 70, 90, 99, and 100% ethanol). Subsequently the brains were cleared and mounted in methyl salicylate (Roth, Karlsruhe, Germany).

### Confocal imaging

Confocal image stacks of the whole brains or brain slices were acquired as described in Zwaka et al. (2016). In short, we used a confocal laser scanning microscope (Leica TCS SP2, Wetzlar, Germany). Sections were scanned at a resolution of 1024 × 1024 voxels each, and a voxel size of 0.61 × 0.61 × 1.3 μm or, 0.73 × 0.73 × 1.1 using a 40 × 0.4 IMM lens objective or a 20 × 0.5 water lens objective. We used light at 488 nm to visualize the neuropil stained with Lucifer yellow and at 633 nm to image the stained neuron. Linear intensity compensation was applied to adjust differences in brightness depending on scanning depth.

### Image and data processing

Images were acquired in Amira (Version 4.1., Mercury Comp, San Diego, CA, USA) and, if necessary, adjusted in size, color contrast and orientation with Adobe R Photoshop R Elements 2.0 (Adobe Systems, Inc., San Jose, CA, USA). For better comparison, some images were mirrored.

### Terminology

The terminology for structural components of the honeybee brain were used according to the nomenclature system of the Insect Brain Name Working Group (Ito et al., 2014). For orientation landmarks prefix “n-” indicates directions that are based on the neuraxis (Ito et al., 2014).

## Results

**Figure 2:**
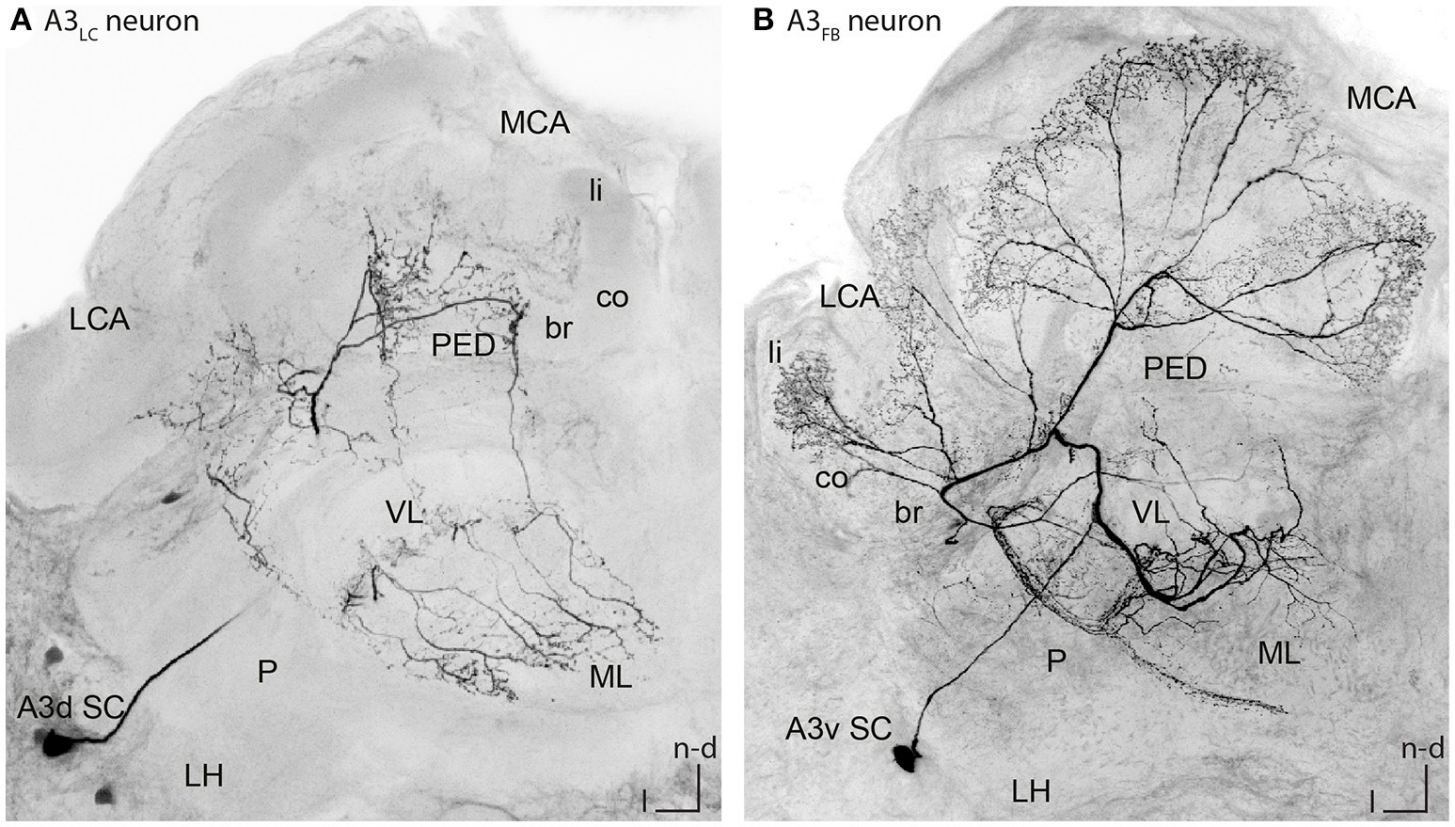
Single stained A3_LC_ and A3_FB_ neurons in the honeybee brain. **(A)** An A3_LC_ neuron that innervated the ventral and the medial lobe. This neuron invaded the peduncle. In the vertical lobe it innervated two divisions, the basal ring and the lip division. In the basal ring division, it exhibited bleb like structures. In the medial lobe, it innervated the basal ring division only. Its cell body laid in the A3 dorsal cluster (compare with Figures 4F, 5C). **(B)** An A3_FB_ neuron that projected from the vertical lobe back to the calyces. In the calyces, it innervated the lip region where it displayed numerous bleb-like structures. In the vertical lobe, it innervated the lip division. Its cell body laid in the A3 ventral cluster (compare Figures 3D, 4I, 5F). A3d SC, dorsal A3 soma cluster; A3v SC, ventral A3 soma cluster; br, basal ring; co, collar; l, lateral; LC, lobe connecting; LCA, lateral calyx; LH, lateral horn; li, lip; MB, mushroom body; MCA, medial calyx; ML, medial lobe; n-an, n-anterior; n-d, n-dorsal; n-an, n-anterior; P, protocerebrum; PED, peduncle; VL, vertical lobe.

In this study, we evaluated the morphology of 31 A3 neurons including nine single stained cells (*n* = 15 animals). Four of these A3 cells were lobe connecting neurons (A3_LC_ neurons, see Figure 2A for example cell and Video S1). The name lobe connecting neurons refers to the fact that these cells connect the medial and the vertical lobe. A3_LC_ neurons did not innervate the calyces but arborized only in the lobes and the peduncle. The remaining five were feedback neurons (A3_FB_ neurons, see Figure 2B for example cell and Video S2), connecting the mushroom body (MB) output region (vertical and medial lobe) with the input region (calyces). The name feedback neurons is adapted from previous studies and refers to this group feeding back information from the mushroombody output area to the mushroombody input area (Gronenberg, 1987; Rybak and Menzel, 1993; Grünewald, 1999b). For a detailed description of the anatomy of all cells see Table S1.

### Characteristics of A3 feedback neurons

**Figure 3:**
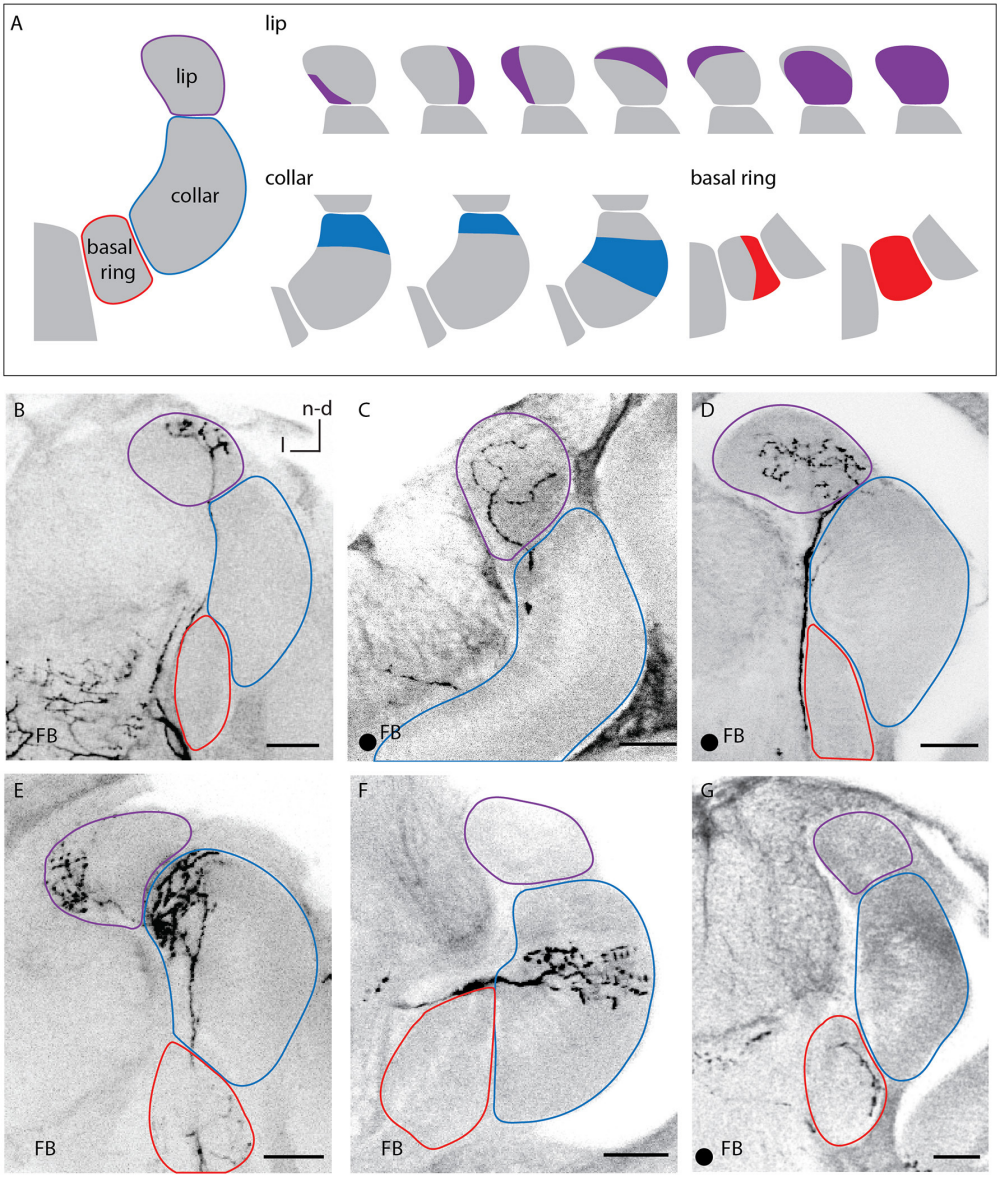
Innervation patterns of A3_FB_ neurons in the calycal subcompartments. **(A)** 1-3 Sketches of the different innervated regions of the lip, the collar, and the basal ring labeled in this study. **(B–G)** Images of labeled feedback neurons. The innervated area remained the same throughout the depth of the circular neuropil of the calyces and was the same for the medial and lateral calyx. The arborisations in the lip were either restricted to different regions of the lip's rim **(B,E)**, homogeneously throughout the lip **(C)** or distributed over a large area with a small non-innervated rim **(D)**. The innervation of the collar was always layered **(E,F)**. The basal ring can either be innervated homogeneously or only be innervated in one area **(G)**. Scale bar = 50 μm. In all panels, dorsal is upwards and lateral is to the left. FB, feedback neuron; l, lateral; n-d, n-dorsal. Projection views of single cell markings are labeled with a black dot in the left corner.

**Figure 4:**
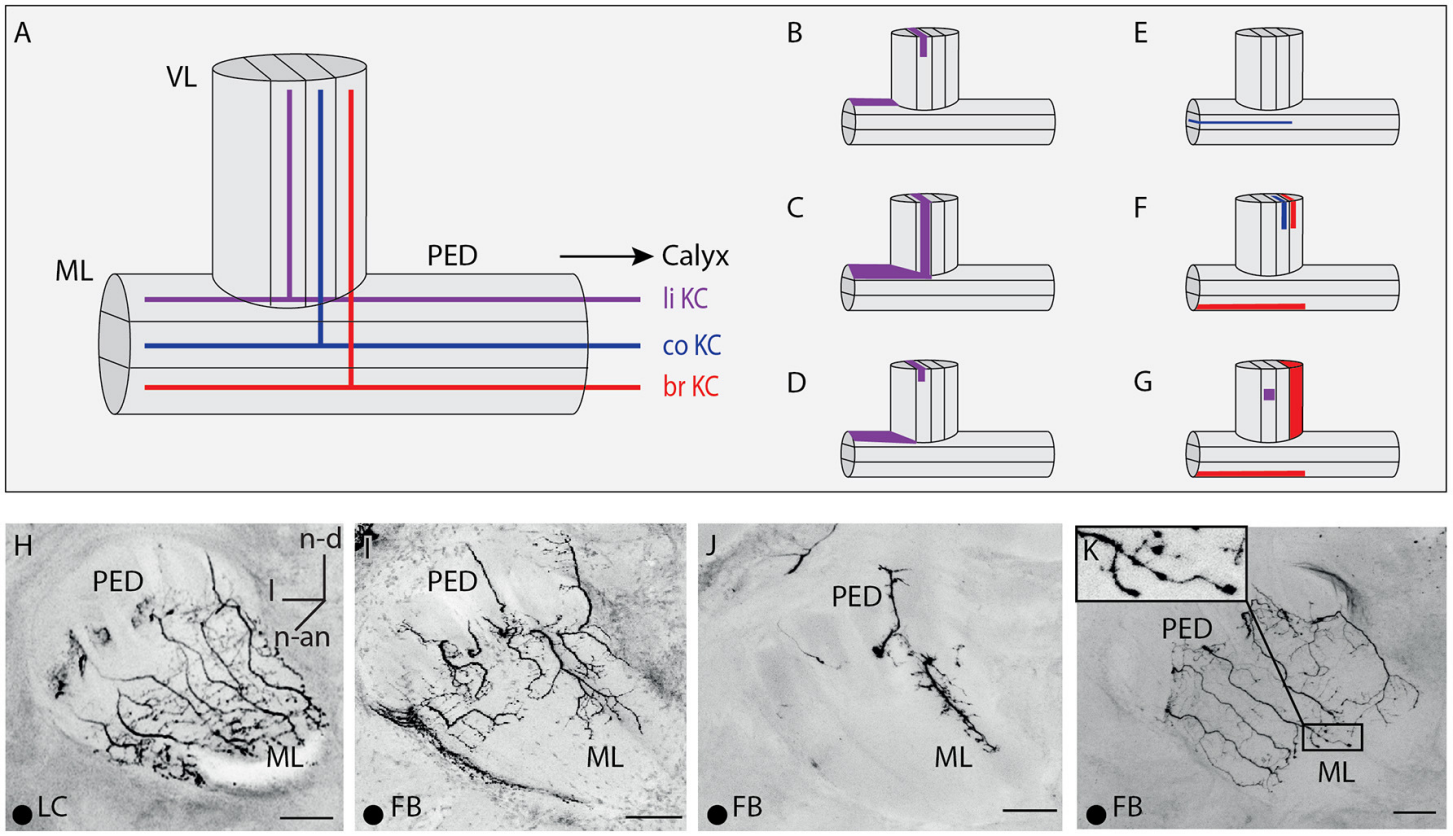
Innervations within the vertical lobe, medial lobe, and the peduncle by A3 neurons. **(A)** Sketch of the vertical and medial lobe and their innervation of different compartments by KCs innervating the lip, collar and basal ring. **(B–G)** Sketches of the vertical and medial lobe as in **(A)**. Highlighted areas illustrate innervation by single A3 neurons in this study. **(H–K)** Innervation pattern of A3 neurons arborizing in the peduncle and the medial lobe. The peduncle is either innervated symmetrically as in **(H,K)** or asymmetrically as in **(I,J)**, with **(J)** innervations only in the medial part of the peduncle. Note that with similar innervation patterns, innervation density can vary. **(H)** An lobe-connecting A3 neuron innervating the medial lobe and the peduncle. **(I)** Asymmetrical innervation in the medial lobe. The same A3_FB_ connects lip divisions in calyx and vertical lobe (compare Figures 3D, 5F). **(J)** Asymmetrical innervation of the medial lobe and the peduncle with a branch only on one side. **(K)** At the most distal parts of the branches, the feedback A3 neuron displayed a few blebs. In all panels, dorsal is upwards and lateral is to the left. br, basal ring; FB, feedback neuron; l, lateral; li, lip; co, collar; LC, lobe connecting neuron; ML, medial lobe; n-an, n-anterior; n-d, n-dorsal; n-an, n-anterior; PED, peduncle. Scale bar = 50 μm. Projection views of single cell markings are labeled with a black dot in the left corner.

**Figure 5:**
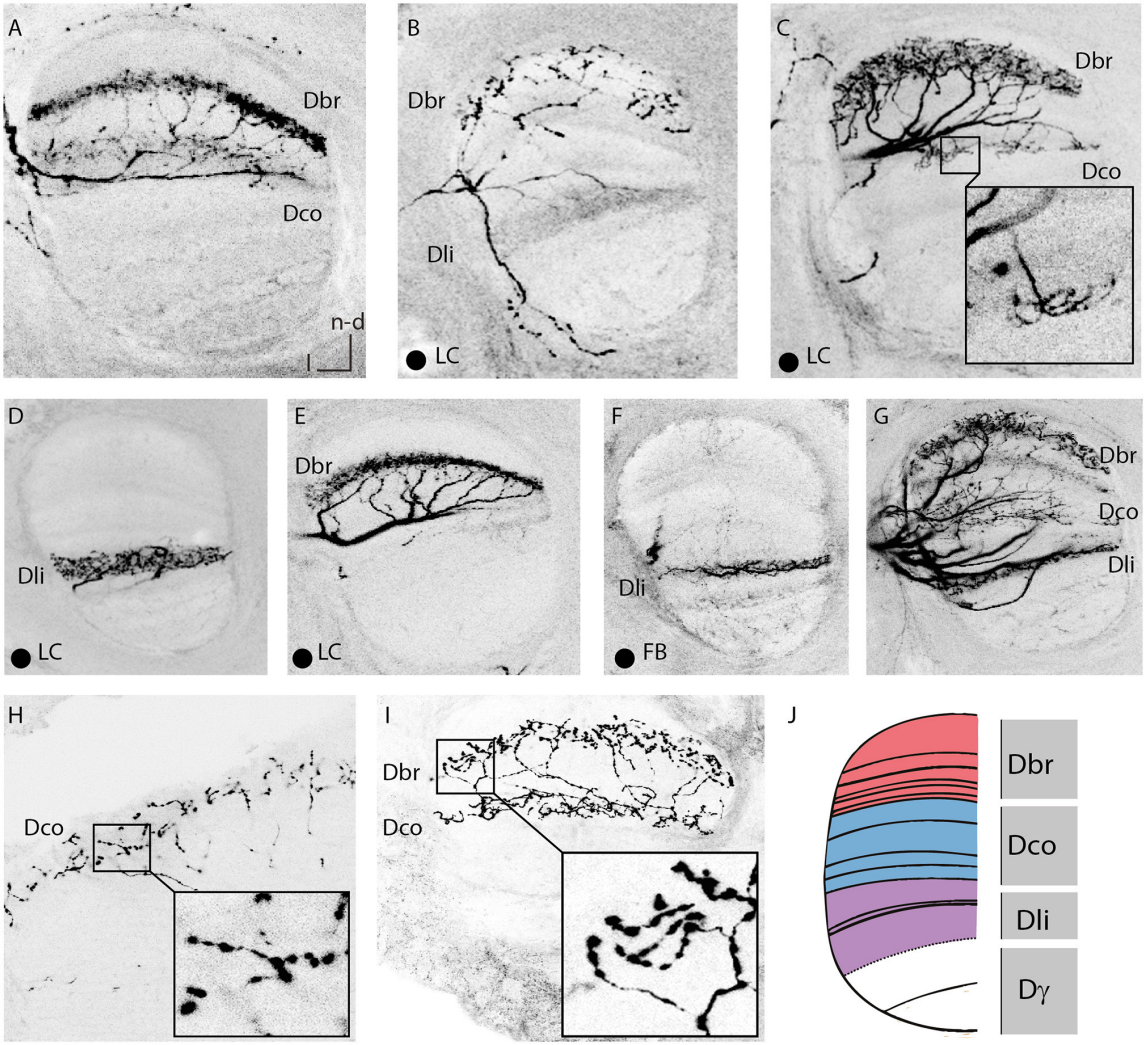
Innervations of the vertical lobe. **(A)** Dense innervation of a thin layer in the in basal ring division of the VL by two A3 neurons. Two somata were stained, one in the A3v and one in the A3d cluster. In the calyx, the A3_FB_ neuron innervated the collar but not the basal ring (compare Figure 3F). **(B)** Sparse innervation of the basal ring division of the VL and the lip division of the VL by an A3_LC_ neuron. This neuron exhibited bleb-like structures in the basal ring division of the VL. **(C)** Dense innervation of a broader layer in the basal ring division of the VL with bleb-like structures by an A3_LC_. The single axon branched and arborized in a thin layer of the collar division of the VL. **(D)** Dense innervation of a lobe connecting neuron in the lip division of the VL. **(E)** Dense innervation of a thin layer in the basal ring division of the VL. **(F)** Sparse innervation of a thin layer of the lip division of the VL by a feedback neuron that arborized in the lip region of the calyx (compare Figure 3D). **(G)** Staining of five A3 neurons of both innervation types that arborized in each division of the VL. **(H)** Arborizations with bleb-like structures in the collar division of the VL by an A3 neuron. Same cells as in Figure 3E. **(I)** Arborisations with blebs in the basal ring division of the VL and with no blebs in the collar division of the VL. Same cells as in **(A)**. **(J)** Sketch of corresponding divisions in the vertical lobe to the calyx subcompartments (adapted after Strausfeld, 2002). Dbr, division corresponding to the basal ring; Dco, division corresponding to the collar; Dli, division corresponding to the lip; Dγ, gamma division of the vertical lobe; FB, feedback neuron; l, lateral; LC, lobe connecting; ML, medial lobe; n-d, n-dorsal; PED, peduncle. Scale bar = 50 μm. Projection views of single cell markings are labeled with a black dot in the left corner. In all panels, dorsal is upwards and lateral is to the left.

Figure 2B illustrates a typical branching pattern of an A3_FB_ neuron (Table S1 cell A3-v1). The soma of the cell lied in the ventral A3 cluster in the lateral protocerebrum. From there the cell sent a branch into the protocerebral tract (PCT) through the peduncle toward the calyces. Close to the alpha-exit of the VL the cell bifurcated and one branch projected toward the lobes and one toward the calyces. In the calyces, this cell furcated again and sent extensions into each calyx. In the lip it exhibited multiple bleb-like structures. It densely innervated the center of the neuropil and left the rim non-innervated (Figure 3D). In the lip division of the medial lobe innervation was asymmetrically: the neuron exhibited branches reaching further into the medial lobe on the medial side than on the lateral side (Figure 4I). In the vertical lobe, this cell innervated a narrow band in the lip division as well (Figure 5F).

In the following we will describe the relevant innervation types of A3_FB_ neurons by MB neuropil.

#### Calyces

A3_FB_ neurons innervated the lip, collar or basal ring of the calyces (Figure 3A). Each neuron branched only in one of the calycal subcompartments. This is consistent with earlier findings (Grünewald, 1999b). The innervated compartment remained the same throughout the depth of the innervated calyx neuropil and it was the same for the medial and lateral calyx. However, we found that the neuropils were not innervated completely: each of the sub- compartments of the calyx could be divided into narrower regions (Figure 3A right side).

The arborisation in the lip was either restricted to different regions of the lip's rim (Figures 3B,E), was homogeneously throughout the whole lip (Figure 3C) or left only a narrow non-innervated rim (Figure 3D right side).

The collar was always innervated by stratified arborisations with varicosities, similar to KCs innervation patterns in the collar that exhibit stratified dendritic trees (Strausfeld, 2002). A3_FB_ neurons described here arborized densely in the outer collar and in a stratum next to the outer collar (Figures 3A,E,F; Video S3).

The basal ring was either fully innervated or partially innervated in subregions of the neuropil (Figures 3A,G).

#### Peduncle

In the peduncle, i.e., the connecting neuropil between the lobes and the calyces, A3_FB_ innervation was very diverse. Neurons showed innervation ranging from sparse to dense. The peduncle is also segmented into divisions by the innervation patterns of KCs descending from the MB calyces (Figure 4A): the most posterior division corresponds to the basal ring, the central division to the collar, and the outer division to the lip region of the calyces (Mobbs, 1982). This segmentation is also found in the medial and vertical lobe (Mobbs, 1982). In the peduncle, A3_FB_ neurons innervated the division that corresponded to the same calycal subcompartment that was innervated in the vertical and medial lobe. For example, an A3_FB_ neuron that projected into the lip where it left only a narrow non-innervated rim arborized in the ring-like outer division of the peduncle (Table S1 cell A3-v1, Figures 4B,I). Kenyon cells that descend from the lip are located in this division. The A3FB cell also innervated the lip division in the medial and vertical lobe as described before.

#### Medial lobe

In the medial lobe, A3_FB_ neurons showed various innervation patterns ranging from medium to dense innervation (Table S1). The innervated divisions in the medial lobe matched innervated division found in the vertical lobe and the calyces as mentioned before (compare Figure 5). The innervations in the medial lobe can be asymmetric in A3_FB_ neurons: We observed broader innervations in the medial than in the lateral area of the medial lobe (Table S1 cell A3-v1, Figure 4I). One cell that innervated the collar region in the calyces displayed innervations restricted to the collar division of the medial lobe (Figure 4E): It exhibited one main branch with multiple fine arborizations restricted to the medial part of the peduncle and the medial lobe (Table S1 cell A3-v3, Figure 4J). Previous data from A3_FB_ neurons indicated that there are cells that invade only the margins of the medial lobe (Grünewald, 1999b).

In addition, we found bleb-like varicosities at A3_FB_ branches innervating the medial lobe (Table S1 cell A3-v2, Figure 4K). The innervation in the lip division of the medial lobe was sparse and symmetric. The same cell sparsely innervated the inner margin in the lip in the calyces and sparsely innervated the lip division of the vertical lobe (Figure 4B).

#### Vertical lobe

A3_FB_ neurons innervated all divisions of the vertical lobe except for its gamma division. Here, the arborisation patterns did vary: Innervations were either thin or broad bands with dense or sparse innervation (Figure 5, Table S1). Previously it was thought that A3 neurons omit the first 30 μm of the vertical lobe (Grünewald, 1999b). Here we found two out of five single stained A3_FB_ cells that cover the first 30 μm below the anterior surface (Figure S1).

All stained A3_FB_ cells with vertical lobe branches innervated divisions that corresponded to the innervated division in the MB calyx. Previously, A3 neurons connecting the basal ring and the collar with their corresponding vertical lobe zones were already known (Grünewald, 1999b). However, a feedback connection to the lip zone in the calyx from the lip division of the vertical lobe as described here was not known before. One cell seemed to entirely lack innervation in the medial lobe (Table S1 cell A3-v4). The same cell innervated a band at the dorsal rim of the basal ring in the calyces where it exhibited very large bleb-like structures (Figure 3G). In addition, it densely innervated the basal ring division of the medial lobe. In two multiple cell staining we found bleb-like varicosities in the basal ring and the collar division of the vertical lobe as well. Many en passant blebs were tightly arranged along these branches (Figures 5H,I).

### Characteristics of A3 lobe connecting neurons

In contrast to A3 feedback neurons, A3 lobe connecting neurons did not innervate the calyces. Figure 2A shows a typical branching pattern of an A3_LC_ neuron. This cell had its soma in the dorsal A3 cluster in the lateral protocerebrum. It sent a branch into the MB. Here it invaded two divisions in the vertical lobe, the basal ring and the collar division. In the basal ring division, it exhibited bleb-like varicosities (Figure 5C). In the medial lobe the neuron only innervated the basal ring division (Figure 4F). In addition, it sent branches into the outer rim of the peduncle (Figure 2A).

#### Peduncle

In the peduncle, A3_LC_ neurons showed varying innervation ranging from sparse to dense (Table S1). Similar to A3_FB_ neurons, A3_LC_ neurons innervated divisions that corresponded to the same calycal subcompartments as in the vertical and medial lobe: For example, an A3_LC_ neuron (Table S1 cell A3-d5) arborized in the lip division of the peduncle and the medial lobe (Figures 4C,H) and densely innervated the lip division of the vertical lobe (Figure 5D). In the medial lobe it showed dense innervation with a few bleb-like structures.

#### Medial lobe

In the medial lobe, like in the peduncle, A3_LC_ neurons showed various innervation patterns ranging from sparse to dense innervation and showed bleb-like varicosities (Table S1). Again, the innervated divisions in the medial lobe matched innervated division found in the vertical lobe.

#### Vertical lobe

A3_LC_ neurons like A3_FB_ neurons omitted the gamma division of the vertical lobe but innervated all other divisions of the vertical lobe. Innervations were either thin or broad bands with dense or sparse innervation (Figure 5). We found that all four single stained A3_LC_ cells covered the first 30 μm below the anterior surface (Figure S1).

We identified two subtypes of A3_LC_ cells: subtype 1 innervated more than one “calyx corresponding” division in the vertical lobe (Figures 5B,C) and subtype 2 innervated only one “calyx corresponding” division in the vertical lobe (Figures 5D,E). One subtype 1 A3_LC_ neuron in this study densely innervated the lip division of the vertical lobe and sparsely innervated the basal ring division (Table S1 cell A3-d4, Figure 5B). In the basal ring division, it displayed bleb-like structures. In the lip division it only innervated a small area (Figure 4G). The same cell sparsely innervated only the basal ring division of the medial lobe. Another subtype 1 A3_LC_ cell arborized within the basal ring division and sparsely innervated the collar division in the vertical lobe (Table S1 cell A3-d3). In the basal ring division, it exhibited a dense innervation with no bleb-like structures and a more medium innervation with bleb-like structures. Each of the collaterals innervating the basal ring division exhibited one to three branches that sparsely innervated the collar division (Figures 4F, 5C). The cell sparsely innervated the basal ring division of the medial lobe where it also displayed bleb-like structures. Thus, both A3_LC_ cells that innervated two areas in the vertical lobe innervated only one area in the medial lobe. In contrast, a subtype 2 cell (Table S1 cell A3-d5, Video S1) innervated only the lip division of the vertical lobe (Figure 5D) and the medial lobe (Figures 4C,H).

## Discussion

The results of our study document that A3 extrinsic neurons of the mushroom body (MB) are a more heterogeneous group than previously assumed. Earlier studies showed that A3 neurons either belong to the A3d (dorsal) cluster and innervate all MB neuropils except for the calyces (here called A3_LC_ neurons) or they belong to the A3v (ventral) cluster and innervate all parts of the MB (here called A3_FB_ neurons) (Rybak and Menzel, 1993; Grünewald, 1999b). Our study confirms these subgroups and refines their anatomy. In addition, it reveals new subtypes within these two major groups that are discussed in the following.

### Similarity between A3_LC_ neurons and A3_FB_ neurons

We describe for the first time the anatomical details of A3_LC_ neurons and show that their innervation in the lobes in some respects are similar to A3 feedback cells: A3_LC_ neurons only innervate one division in the medial lobe and connect to the same calyx-corresponding division in the vertical lobe, which is the main output region of the MB. Both, vertical lobe and medial lobe, consist of subdivisions that are defined by the axons of KCs descending from the lip, basal ring, or collar of the calyces (Mobbs, 1982; Strausfeld, 2002).

### A3_LC_ neurons include two subtypes

We identified to two sub-types of lobe connecting neurons (A3_LC_): Subtype 1 innervated two different divisions in the vertical lobe and exhibited bleb-like structures in one of the divisions (Figures 5B,C), subtype 2 formed dendritic innervations in various depths in one division in the vertical lobe (Figures 5D,E). Subtype 1 cells found in this study exhibited bleb-like varicosities in the basal ring division of the vertical lobe and innervated the basal ring division of the medial lobe. In addition, they innervated a second division in the vertical lobe. Bleb-like varicosities are thought to be mostly presynaptic (Ganeshina and Menzel, 2001) and thus indicate a putative output region in the vertical lobe in addition to the medial lobe. It is unclear whether subtype 1 neurons connect the output of different or the same modality in the vertical lobe as collar, basal ring and lip all get multimodal input (Mobbs, 1982; Abel et al., 2001; Gronenberg, 2001; Zwaka et al., 2016). However, this might possibly allow for across modal processing in the vertical lobe. Subtype 2 might represent an inhibitory connection from vertical lobe (input region) to the medial lobe (output region) within the same modality or for different modalities from the same calycal subdivision. Earlier studies already indicated that in the medial and vertical lobe local connections between KCs type 1 and inhibitory extrinsic neurons exist (Grünewald, 1999b).

### A3_FB_ neurons innervate calycal subcompartments

A given A3_FB_ neuron innervates only one of the three calycal subcompartments lip, collar, and basal ring (Grünewald, 1999b). In contrast to earlier findings, we found that the innervation areas do not cover the complete calycal zones. Each of the subcompartments lip, basal ring, and collar can be divided into narrower regions. Our confocal data revealed that A3_FB_ (A3v) target specific smaller regions of the respective calyx subcompartments (Figure 3). This specificity in the innervation region suggests a complex, target specific inhibitory network rather different from Locust and Drosophila. In these latter species, only one GABAergic feedback neuron provides inhibition most likely for a general sparse coding scheme in KCs (Lei et al., 2013; Lin et al., 2014) since the anterior paired lateral neuron (APL) inhibits KCs in an all-to-all fashion (Lin et al., 2014). However, a recent study suggests that this single neuron can also exert local inhibition on specific KCs (Inada et al., 2017). Contrarily, in honeybees, about 50 inhibitory A3_FB_ appear to serve specific target networks (Grünewald, 1999b).

Zones within the calycal subcompartments have been characterized by their innervation areas of KCs and their different immunoreactivity to FMRFamides (Strausfeld, 2002). Some A3_FB_ innervation patterns described here resemble the zones described by Strausfeld. Other innervated areas do not fit into these zones indicating additional networks. None of the A3_FB_ neurons involved match the described regions completely (Figure 3).

### Feedback systems in the insect calyces

In cockroaches, four giant GABAergic neurons supply feedback information onto the MB calyces (Weiss, 1974; Yamazaki et al., 1998), whereas in the bee about 50 of these neurons exist (Grünewald, 1999b). The cockroach neurons receive input from other MB output neurons (Weiss, 1974; Nishino and Mizunami, 1998; Yamazaki et al., 1998; Strausfeld and Li, 1999) that are not known in more detail. The innervated subdivisions in the calyces match the zonation made by axon terminals from neurons connecting the antennal lobe and the calyces (Takahashi et al., 2017). Earlier studies showed that GABAergic feedback neurons in honeybees form putative synaptic contacts with projection neurons that connect the antennal lobe with the MB. Here, projection neuron presynapses form microcircuits with KCs, A3_FB_ neurons, and with tentative modulatory neurons, most likely the octopaminergic VUM neuron (Ganeshina and Menzel, 2001; Zwaka et al., 2016). In Drosophila larvae, the APL neuron synapse onto KCs but also forms few contacts with projection neurons (Masuda-Nakagawa et al., 2014). In adult flies, KCs are receiving inhibition from GABAergic cells in the calyces as well (Yasuyama et al., 2002), most likely from the APL neuron.

### Innervation within “calyx corresponding” divisions

Most A3FB neurons found in this study stay within one “calyx corresponding” division throughout the brain. In the peduncle, A3 neurons innervated divisions corresponding to the same calycal subcompartment as in the vertical lobe and the medial lobe, supporting the conclusion that feedback connections stay within higher order sensory processing areas. Along their path through the peduncle, A3 neurons might collect information from certain KC populations (Grünewald, 1999b).

Previous studies suggested that A3_FB_ neurons mostly connect zones of the vertical lobe with the corresponding subcompartments of the calyces (Grünewald, 1999b). We can show that this is also true in the medial lobe: Here A3_FB_ neurons arborize only in zones that correspond to the calycal subcompartments that the same cells are innervating. In addition to the previously described types of A3_FB_ neurons F1-F4 (Grünewald, 1999b), we found cells that connect the lip region in the MB with the lip division of the vertical and medial lobe (Figure 1).

### Asymmetric innervation of the medial lobe

The innervation areas within the medial lobes may be asymmetric with varying innervation densities. The medial lobes are divided in a lateral and a medial half. KCs of the lateral calyx project into the lateral half and KCs of the medial calyx into the medial half (Mobbs, 1982). This different innervation of A3_FB_ neurons suggests that some neurons get only input from one of the two calyces whereas other get input from both. Thus, this indicates different processing of information from the medial and lateral calyx at the level of the medial lobe.

### Putative output sides in the lobes

A3_FB_ cells in this study showed bleb-like varicosities in the medial lobe indicating putative output sides additionally to the calyces. In multiple cell staining we found bleb-like varicosities in the vertical lobe as well. It is unclear whether these belonged to A3_FB_ neurons or whether they are output regions of A3_LC_ neurons as discussed above. Similar, in *Drosophila*, the APL neuron displays input and output sides in the alpha lobe (Takemura et al., 2017). Here, it mostly connects to KCs and is thus implied to mainly influence the sensory information that is conveyed to the lobes (Takemura et al., 2017). In *Schistocerca americana* synapses between a GABAergic neuron and KCs were found both in the peduncle and the lobe. This single “giant GABAergic neuron” (GGN) innervates each side of the MB (Leitch and Laurent, 1996). It forms dendrites in the vertical lobe and peduncle and axons in the calyces (Watson and Burrows, 1985) suggesting an inhibitory feedback that might be similar to the A3_FB_ neurons described here and the APL in *Drosophila* (Liu and Davis, 2009; Papadopoulou et al., 2011).

### Role of GABAergic feedback in the insect brain

In the honeybee MB, no direct evidence exists to date for the role of GABAergic feedback neurons. Pharmacological experiments suggest some role in non-elemental forms of olfactory learning (Devaud et al., 2015) and possibly also in reversal learning. In the latter case ionotropic GABAergic signaling from the lobes to the calyces seems to be required for the reversal of the stimulus valence (Boitard et al., 2015). A3 neurons in the honeybee were found to change their response properties specifically and selectively in context dependent forms of learning. This suggests that they are involved in attention selection mechanisms (Filla and Menzel, 2015). The APL neuron in *Drosophila* is also involved in learning and memory. It facilitates reversal learning (Wu et al., 2012) and suppresses olfactory learning and in turn its activity is suppressed by olfactory learning (Liu and Davis, 2009). In addition, the APL neuron appears to be involved in decorrelating and sparsening KC signaling leading to increased odor discrimination (Lin et al., 2014). In honeybees, data suggests that sparse odor coding at the level of KC might be partially dependent on GABAergic gain control between projection neurons and KCs. Moreover, sparse coding seems to depend on an additional GABA-independent effect that leads to a shorter ON response in KCs (Farkhooi et al., 2013; Froese et al., 2014). In locusts, the non-spiking inhibitory feedback neuron connects the output of KCs with the input and is hypothesized to control the excitability of KC maintaining sparse odor coding (Papadopoulou et al., 2011).

In summary, it appears that the honeybee has the most advanced inhibitory feedback system of all insects investigated so far. It is plausible that this elaborated system is required for proper functioning of multiple target specific subnetworks of the honeybee MB.

## Author contributions

HZ: conception and design, analysis and interpretation, writing the article. RB: conception and design, acquisition of data, analysis and interpretation of data, writing the article. BG: revising the article. RM: conception and design, revising the article.

### Conflict of interest statement

The authors declare that the research was conducted in the absence of any commercial or financial relationships that could be construed as a potential conflict of interest.

## Supplementary material

All original scans of cells reported in this study are available at: https://insectbraindb.org/neurons/group/220086ec-b5b7-46f4-9781-e521510f143b.

Figure S1 A3 neuron innervation of the mushroom body. This orthogonal view of A3 innervation shows arborisation of an A3 neuron in the anterior part of the vertical lobe. Note the innervation of the vertical lobe in the first 30 μm from n-anterior. Lines indicate the respective projection plane XY, YZ or XZ. LCA, lateral calyx; MCA, medial calyx; VL, vertical lobe. Prefix “n-” indicates directions that are based on the neuraxis (Ito et al., 2014). Scale bar = 100 μm.

Table S1 Table includes all A3 neurons stained in this study. Detailed descriptions of each staining includes name in the insect brain data base, figures that show the staining, and anatomical details of the cells. Single-cell staining are indicated compared to multi-cell staining. The location of the soma in the dorsal or ventral cluster is noted. Innervation area and density in the calyces and lobes are described. Staining that remained inconclusive are marked.
