# Supplementary figures and images for: Neural Organization of A3 Mushroom Body Extrinsic Neurons in the Honeybee Brain
Source: Front Neuroanat. 2018 Aug 3;12:57. doi: 10.3389/fnana.2018.00057 (PMC6089341; doi:10.3389/fnana.2018.00057)

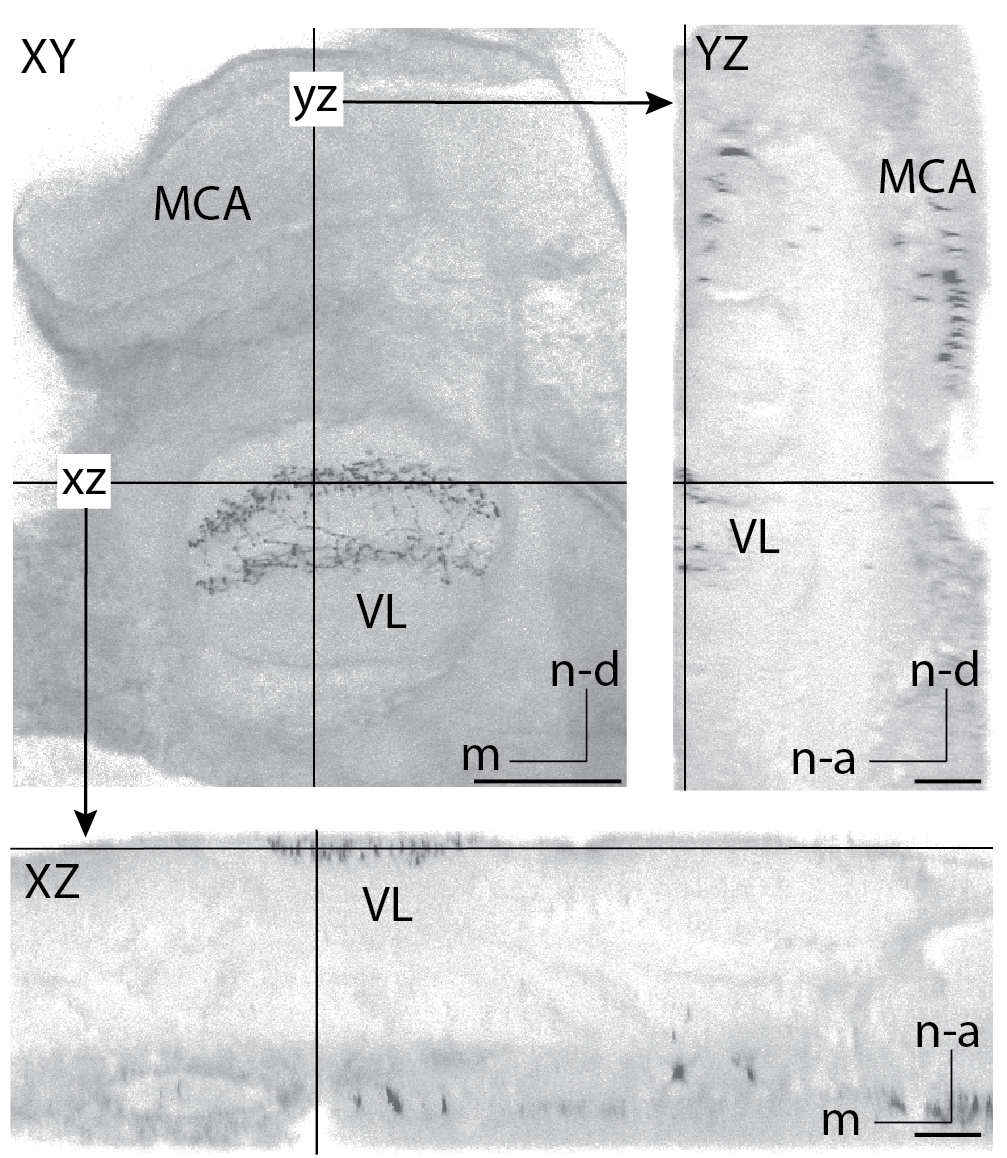

Supplement: Figure S1 — A3 neuron innervation of the mushroom body. This orthogonal view of A3 innervation shows arborisation of an A3 neuron in the anterior part of the vertical lobe. Note the innervation of the vertical lobe in the first 30 μm from n-anterior. Lines indicate the respective projection plane XY, YZ or XZ. LCA, lateral calyx; MCA, medial calyx; VL, vertical lobe. Prefix “n-” indicates directions that are based on the neuraxis (Ito et al., 2014). Scale bar = 100 μm. [file Image_1.TIF]
