# Supplementary material for: Neural Organization of A3 Mushroom Body Extrinsic Neurons in the Honeybee Brain
Source: Front Neuroanat. 2018 Aug 3;12:57. doi: 10.3389/fnana.2018.00057 (PMC6089341; doi:10.3389/fnana.2018.00057)
Supplement: Table S1 — Table includes all A3 neurons stained in this study. Detailed descriptions of each staining includes name in the insect brain data base, figures that show the staining, and anatomical details of the cells. Single-cell staining are indicated compared to multi-cell staining. The location of the soma in the dorsal or ventral cluster is noted. Innervation area and density in the calyces and lobes are described. Staining that remained inconclusive are marked. [file Table_1.pdf]

insectbraindb.org	Name in paper	Figure number	Single cell	Soma cluster	Mushroom body calyx innervation			Medial lobe innervation			Vertical lobe innervation			
					lip	co	br	lip division	co division	br division	innervation first 30µm n-anterior	lip division	co division	br division
A3-d2	$A3_{LC}1$	5E	yes	d	no	no	no	n.d.	n.d.	n.d.	yes	no	no	narrow, dense innervation
A3-d3	$A3_{LC}2$	2A, 4F, 5C mirrored	yes	d	no	no	no	no	no	sparse innervation with blebs	yes	no	sparse innervation	medium innervation (with blebs) and dense innervation (no blebs)
A3-d4	$A3_{LC}3$	4G, 5B mirrored	yes	d	no	no	no	no	no	sparse innervation possibly with blebs	yes	dense innervation	no	broad, sparse innervation, with blebs
A3-d5	$A3_{LC}4$	4C, 4H, 5D, video S1	yes	d	no	no	no	dense innervation with few blebs	no	no	yes	dense innervation	no	no
A3-v1	$A3_{FB}2$	2B, 3D mirrored, 4B, 4I, 5F, video S2	yes	v	centrally, dense innervation	no	no	asymmetric, medium innervation	no	no	no	narrow, medium innervation	no	no
A3-v2	$A3_{FB}3$	4B,4K	yes	v	inner margin, sparse innervation	no	no	sparse innervation with blebs	no	no	no	narrow, sparse innervation	no	no
A3-v3	$A3_{FB}4$	4E, 4J mirrored	yes	v	no	outer collar, medium innervation	no	no	asymmetric, medium innervation	no	yes	n.d.	n.d.	n.d.
A3-v4	$A3_{FB}5$	3G mirrored	yes	v	no	no	dorsal, medium innervation, very large blebs	no	no	no	no	no	no	narrow, dense innervation
A3-v5	$A3_{FB}6$	3C mirrored,4D	yes	v	fully, sparse innervation	no	no	yes, npe	n.d.	n.d.	n.d.	sparse innervation	no	no
A3-1	A3-1	3F, 5A mirrored, 5I, S1, video S3	no	1v,1d soma	no	centrally, dense innervation	no	n.d.	n.d.	n.d.	yes	no	yes	with blebs
A3-2	A3-2	--	no	several soma	n.d.	n.d.	n.d.	sparse innervation	n.d.	sparse innervation	broad, dense innervation	n.d.	n.d.	n.d.
A3-3	A3-3	3E mirrored, 5H	no	3v, 1 d	inner margin, medium	outer collar, medium innervation	no	n.d.	n.d.	n.d.	yes	n.d.	dense innervation, with blebs	n.d.
A3-4	A3-4	--	no	4v,3d soma	no	outer collar, sparse innervation	fully, sparse innervation	n.d.	sparse innervation	sparse innervation	yes	n.d.	n.d.	yes, npe
A3-5	A3-5	3B, 5G mirrored	no	5 soma	outer margin, sparse innervation	no	no	medium innervation	asymmetric, sparse innervation	medium innervation	yes	medium to dense innervation	medium innervation with	sparse innervation to dense with blebs
A3-6	A3-6	--	no	2 soma	outer margin, sparse innervation	no	no	dense innervation	no	no	n.d.	yes, npe	no	no

n.d. = not determinable; npe = not possible to evaluate innervation density(
